# Elevated Aspartate and Alanine Aminotransferase Levels and Natural Death among Patients with Methamphetamine Dependence

**DOI:** 10.1371/journal.pone.0029325

**Published:** 2012-01-05

**Authors:** Chian-Jue Kuo, Shang-Ying Tsai, Ya-Tang Liao, Yeates Conwell, Wen-Chung Lee, Ming-Chyi Huang, Shih-Ku Lin, Chiao-Chicy Chen, Wei J. Chen

**Affiliations:** 1 Institute of Epidemiology and Preventive Medicine, College of Public Health, National Taiwan University, Taipei, Taiwan; 2 Department of General Psychiatry, Taipei City Psychiatric Center, Taipei, Taiwan; 3 Department of Psychiatry, School of Medicine, Taipei Medical University, Taipei, Taiwan; 4 Center for the Study and Prevention of Suicide, University of Rochester Medical Center, Rochester, New York, United States of America; Charité-Universitätsmedizin Berlin, Germany

## Abstract

**Background:**

Methamphetamine is one of the fastest growing illicit drugs worldwide, causing multiple organ damage and excessive natural deaths. The authors aimed to identify potential laboratory indices and clinical characteristics associated with natural death through a two-phase study.

**Methods:**

Methamphetamine-dependent patients (n = 1,254) admitted to a psychiatric center in Taiwan between 1990 and 2007 were linked with a national mortality database for causes of death. Forty-eight subjects died of natural causes, and were defined as the case subjects. A time-efficient sex- and age-matched nested case-control study derived from the cohort was conducted first to explore the potential factors associated with natural death through a time-consuming standardized review of medical records. Then the identified potential factors were evaluated in the whole cohort to validate the findings.

**Results:**

In phase I, several potential factors associated with natural death were identified, including aspartate aminotransferase (AST), alanine aminotransferase (ALT), comorbid alcohol use disorder, and the prescription of antipsychotic drugs. In phase II, these factors were confirmed in the whole cohort using survival analysis. For the characteristics at the latest hospital admission, Cox proportional hazards models showed that the adjusted hazard ratios for natural death were 6.75 (*p*<0.001) in the group with markedly elevated AST (>80 U/L) and 2.66 (*p*<0.05) in the group with mildly elevated AST (40–80 U/L), with reference to the control group (<40 U/L). As for ALT, the adjusted hazard ratios were 5.41 (*p*<0.001), and 1.44 (*p*>0.05). Comorbid alcohol use disorder was associated with an increased risk of natural death, whereas administration of antipsychotic drugs was not associated with lowered risk.

**Conclusions:**

This study highlights the necessity of intensive follow-up for those with elevated AST and ALT levels and comorbid alcohol use disorder for preventing excessive natural deaths.

## Introduction

The use of methamphetamine has become one of the fastest growing illicit drug problems worldwide since 1990s, particular in South-east Asia, Australia, and North America [1]. Methamphetamine is now spreading easily, supplied by makeshift-scale (‘kitchen’ and ‘living room’) and industrial-scale clandestine laboratories, where it is often produced using legally purchased ingredients [2]. Furthermore, psychiatric or medical staff members were more likely to care for patients with methamphetamine-related problems in the past decade, and there is emerging literature on the damages associated with regular methamphetamine use, including physical and mental health problems [3], and premature death [4], [5], [6]. As a basis for prevention efforts, however, studies on the factors associated with natural death in this population are limited.

Natural death can result from the comorbid illnesses (e.g., alcoholism, hepatitis virus types B and C) that methamphetamine users are likely to have [3], [7], or from the harmful effects directly attributable to methamphetamine [3], [8]. Regarding the latter, cardiovascular pathology is a well-known toxic effect of methamphetamine, and potential complications include tachycardia, arrhythmia, increased blood pressure, myocardial infarction, and even sudden cardiac death [8]. Additionally, methamphetamine use can induce complications in several major organ systems, including the central nervous system, kidneys, hepatic system, and musculature [3]. To monitor the damage to these organ systems in patients with methamphetamine dependence, routine laboratory testing is often applied in regular clinical practice by using markers reflecting the functions of multiple organs (e.g., aspartate aminotransferase for heart, muscle, and liver) or single specific organs (e.g., thyroxine for thyroid function) [9]. We hypothesized that laboratory indices related to organ systems damage could be proxies in association with natural death.

For exploring the laboratory indices and other clinical characteristics (e.g., comorbid illnesses, prescribed psychiatric drugs) in association with methamphetamine-related natural death, we conducted a two-phase study using a cohort with methamphetamine dependence monitored for a long-term period. Due to unavailability of risk factors for natural death in prior studies, we first conducted a time-efficient nested case-control study derived from the cohort to explore the potential factors associated with natural death. We then evaluated the identified potential factors in the whole cohort to validate the findings. The study aimed to identify potential laboratory indices as surrogate markers for natural death, along with the clinical characteristics associated with natural death.

## Methods

### Setting and Patients

The source of subjects was the Taipei City Psychiatric Center, a psychiatric service center designated for northern Taiwan. The methodology used is described extensively elsewhere and was used in research on suicide mortality among methamphetamine dependents [4]. Briefly, an initial series of 1,548 patients with methamphetamine-related problems admitted to the psychiatric center from January 1, 1990 to December 31, 2007 were retrospectively enrolled. Following the practice of previous studies [4], [10], we then applied strict inclusion criteria based on the principal psychiatric diagnosis of methamphetamine dependence according to the Diagnostic and Statistical Manual of Mental Disorders, Third Edition Revised (DSM-III-R) and Fourth Edition (DSM-IV), which excluded opportunistic users who had used methamphetamine less than 20 times in the past year. The use pattern of methamphetamine for each subject was obtained through careful clinical, semi-structured interviews conducted by a resident psychiatrist and a board-certificated psychiatrist during hospitalization. Comorbidity of alcohol or other substance use disorders for each subject was pertinent to the patients' clinical treatment and carefully diagnosed by the psychiatrists.

Furthermore, to investigate the effect from methamphetamine without being confounded by other substances, we excluded any patient whose principal diagnosis of methamphetamine dependence was changed to another substance use disorder(s) after the index admission. Finally, 1,254 methamphetamine-dependent subjects were enrolled as the cohort.

By using a national identity number as the subject identifier, the cohort was electronically linked with data files held in the Taiwan Department of Health Death Certification System between January 1, 1990 and December 31, 2007, and 130 deaths were identified. The linkage process was approved by the Department of Health in Taiwan [11]. Of the deceased, 48 patients who died natural deaths were selected as the case subjects. The remainder (n = 82) died from unnatural causes.

### Phase I: Nested case-control study

We first conducted a nested case-control study derived from the defined cohort (N = 1,254) to explore the potential risk factors associated with methamphetamine dependence and natural death by means of a time-consuming and comprehensive chart review process. Based on risk-set sampling, each case was matched with four controls or fewer if four suitable controls could not be identified. Matched for age (±5 years), sex, and the year of index admission, controls were selected randomly from the cohort subjects who were alive at the time of death of the case subject. Index admission was defined as the earliest hospitalization during the study period. Among the 1254 subjects, 358 had multiple hospitalizations, but the proportion did not differ between cases with natural death and others. No suitable control was found for three cases, so that a total of 45 case-controlled pairs (i.e., 45 cases and 167 controls, n = 212) were included in the study. Of the pairs, four controls were used in each case of 36 pairs, three controls in each case of six pairs, two controls in each case of two pairs, and one control in one pair.

Semi-structured case notes [12] were systematically recorded for any patient who was admitted to the source hospital and included information on demographic characteristics and psychiatric history, details of the patient's mental status, physical condition, alcohol/drug use disorders, and family history. A parallel interview to confirm the information with family members or others who knew the patient well was routinely conducted during the admission. Psychiatric drugs (e.g., antipsychotic drugs, mood stabilizers, and antidepressants) were prescribed when clinically indicated. On the first morning after admission, a fasting venous blood sample was routinely drawn for biochemical analyses.

Information for each subject was carefully checked using a combined standardized review process by two trained clinical psychologists and then double-checked by a senior psychiatrist (CJK). All of the chart reviewers were blinded as to the subjects' case or control status. In order to facilitate the chart review, a structured abstraction form containing 125 items was developed that typically required at least 1 hour to complete, including information relating to the index and latest hospital admissions during the study period on demographics, social support network, substance use history, symptom profile, prescription of psychiatric drugs, and laboratory data.

Before the review process, all of the chart reviewers participated in a reliability study and rated information independently for four cases and eight controls. The results showed satisfactory inter-rater reliability, with the kappa values of key variables all greater than 0.7, including symptom profiles and comorbid alcohol use disorders.

For statistical analyses, group comparisons between cases and controls were performed using univariate conditional logistic regression analyses initially, and then the variables with a reasonable association with natural death (*p*<0.05) were entered into the multivariable regression analyses.

### Phase II: Cohort study

For those variables identified as factors associated with the risk of natural death from phase I study (reported later), the information was then retrieved for those subjects not included in the nested case-control study. Data collection took about 10 minutes for each subject by means of a brief chart review process. The whole cohort was then analyzed using Cox proportional hazards analyses to validate the results and precisely estimate the risks of natural death based on the identified risk factors.

All of the multivariable models were conducted using SAS software, version 9.2 (SAS Institutes Inc., Cary, NC, USA). A *p* value of 0.05 was considered significant in the multivariable regression analyses.

### Ethics Statement

This study was approved by the Institutional Review Board of the Committee on Human Subjects of Taipei City Hospital. The data were collected based on a retrospective medical chart review process in the fully-restricted medical records room. A waiver was granted for informed consent due to the minimal risk to the privacy of individual subjects and that the identities of subjects were fully encrypted to preserve anonymity during the statistical analysis.

## Results

### Demographics and Drug Use History

Both the deceased patients and the controls among the methamphetamine dependents in this study had similar distributions of age at first methamphetamine use, marital status, living with family, educational level, employment, and socio-economic status based on Hollingshead's classification [13] (**Table 1**).

**Table 1:** Socio-demographic characteristics of natural death and living controls (1∶4 ratio) among patients with methamphetamine dependence at the index admission.

Characteristic	Deceased patients (N = 45)	Living controls (N = 167)	Unadjusted odds ratio	95% confidence interval
	n (%)	n (%)		
Male	41 (91.1)	151 (90.4)	*	*
Being married	11 (24.4)	33 (19.8)	1.27	0.55–2.91
Living with family	38 (86.4)	144 (87.8)	0.77	0.26–2.25
Education ≥12 years	14 (31.1)	70 (42.2)	0.63	0.31–1.25
Employment (past 1 year)	23 (51.1)	95 (56.9)	0.77	0.38–1.55
Hollingshead socio-economic class IV or V (past 1 year)	40 (88.9)	144 (87.3)	1.15	0.41–3.22

	Mean (SD)	Mean (SD)		
Age (years) at index admission, mean (SD)	30.7 (7.8)	29.4 (7.9)	1.06	0.97–1.15
Age at first methamphetamine use (years), mean (SD)	24.0 (8.3)	24.7 (6.9)	0.97	0.88–1.07
Duration of methamphetamine use (years), mean (SD)	5.4 (4.0)	4.7 (3.5)	1.04	0.94–1.15

*Matched by design.

Among the 48 methamphetamine dependent patients who died from natural causes, the mean interval from the index admission to death was 5.3 (SD = 4.0) years. The causes of death included cardiovascular (n = 12), respiratory (n = 3), hepatic (n = 8), cerebrovascular (n = 2), neoplasm (n = 4), endocrine and metabolic (n = 3), genitourinary (n = 1), neurological (n = 1), shock without mention of trauma (n = 1), sudden death with unknown cause (n = 1; ICD-9 code: 798), and other unknown and unspecified causes (n = 12; ICD-9 code: 799.9).

### Clinical Characteristics at the Index and Latest Admissions

The clinical characteristics at the index and latest admissions were compared between cases and controls in **Table 2**. Among the comorbidities examined, a greater proportion of the deceased patients had alcohol use disorders than did the controls. Among the laboratory markers listed in the table, deceased patients had significantly higher mean levels of AST and ALT. The findings in the latest admission were similar. A higher proportion of case patients had alcohol use disorders at the latest admission than did the controls, and also greater AST and ALT levels as well. Additionally, the case patients were less likely to receive antipsychotic drugs at the latest admission than controls (46.7% vs. 67.5%, *p* = 0.009) (**Table 2**). There was no difference between cases and controls in terms of other laboratory indices, including electrolytes (sodium, potassium, chloride), fasting glucose, renal function (blood urea nitrogen, creatinine), uric acid, albumin, globulin, total protein, thyroxine, or platelet count (data not shown).

**Table 2:** Clinical characteristics of patients with methamphetamine dependence dying from natural causes and living controls with methamphetamine dependence at the index admission and the latest admission using univariate conditional logistic regression.

Characteristic	Deceased patients(N = 45)	Living controls (N = 167)	Unadjusted Odds ratio	95% confidence interval
**Index admission**	n (%)	n (%)		
With psychosis	32 (71.1)	115 (68.9)	1.16	0.54–2.49
Co-morbidity				
Alcohol use disorders	9 (20.0)	11 (6.6)	3.63**	1.37–9.58
Other substance disorders	12 (26.7)	29 (17.4)	1.76	0.77–4.01
Schizophrenia	4 (8.9)	23 (13.8)	0.61	0.20–1.88
Psychosis-related symptoms	30 (66.7)	125 (74.9)	0.68	0.33–1.41
Auditory hallucination	23 (51.1)	100 (59.9)	0.69	0.36–1.35
Visual hallucination	13 (28.9)	37 (22.2)	1.40	0.68–2.90
Persecutory delusion	19 (42.2)	82 (49.1)	0.78	0.38–1.60
Reference delusion	16 (35.6)	54 (32.3)	1.19	0.58–2.44
Discharged against medical advice	14 (31.1)	62 (37.1)	0.80	0.40–1.60
Antidepressant use	11 (24.4)	26 (15.6)	1.68	0.73–3.88
Benzodiazepine use	33 (73.3)	114 (68.7)	1.23	0.58–2.60
Mood stabilizer	0 (0.0)	5 (3.0)	0.00	0.00–
Antipsychotic drug use	22 (48.9)	101 (60.5)	0.60	0.29–1.22
Laboratory markers	Mean (SD)	Mean (SD)		
Blood hemoglobin (g/dL)	14.9 (4.4)	14.7 (1.7)	1.03	0.91–1.16
Leukocytes (10^3^ *µ/L)	6.9 (2.9)	7.4 (2.1)	1.00	1.00–1.00
Serum AST (U/L)	84.9 (134.7)	29.2 (25.8)	1.02***	1.01–1.03
Serum ALT (U/L)	88.2 (143.6)	34.7 (55.1)	1.01**	1.00–1.01
Serum cholesterol (mg/dL)	172.1 (41.6)	179.3 (45.2)	1.00	0.99–1.01
Serum triglyceride (mg/dL)	119.8 (67.7)	124.3 (84.7)	1.00	0.99–1.01
Hepatitis B surface antigen, n (%)	10 (23.3)	28 (19.0)	1.23	0.55–2.72
**Latest admission** a				
Alcohol use disorders, n (%)	11 (24.4)	21 (12.6)	2.38*	1.02–5.55
Antipsychotic drug use, n (%)	21 (46.7)	112 (67.5)	0.38**	0.18–0.78
Serum AST (U/L), mean (SD)	87.4 (136.2)	29.0 (29.9)	1.03***	1.01–1.05
Serum ALT (U/L), mean (SD)	91.9 (143.4)	31.9 (44.0)	1.02***	1.01–1.03
Hepatitis B surface antigen, n (%)	10 (24.4)	29 (19.9)	1.19	0.53–2.67

a Only variables at latest admission with *p*<0.05 based on univariate conditional logistic regression and hepatitis B surface antigen are included.

*p<0.05,

**p<0.01,

***p<0.001.

The proportions of underlying physical illnesses requiring clinical attention in case and control subjects at the index admission were 6.7% (3/45) vs. 3.0% (5/162) (*p* = 0.251) for cardiovascular disease, 6.7% (3/45) vs. 1.2% (2/162) (*p* = 0.065) for endocrine disease, 15.6% (7/45) vs. 6.0% (10/167) (*p* = 0.074) for gastrointestinal disease, 6.7% (3/45) vs. 5.4% (9/162) (*p* = 0.721) for genitourinary disease, 4.4% (2/45) vs. 2.4% (4/162) for hematological disease, and 13.3% (6/35) vs. 6.0% (10/167) (*p* = 0.181) for hepatic disease. There were no statistical differences between two groups.

Regarding family history of psychiatric disorders (including schizophrenia, substance use disorders, and suicide), no difference between case and control subjects was found (data not shown).

To conduct the multivariable analyses, we used the variables at the latest admission due to the inclusion of more variables with statistical significance than at the index admission, along with shorter periods between the baseline and the censored point, which improved predictive power.

### Multivariable Analyses

Multivariable conditional logistic regression analyses comprised the variables with significant associations (*p*<0.05), including AST, ALT, antipsychotic drug use, and comorbid alcohol use disorder. Multivariable analyses were analyzed based on AST and ALT separately due to high correlation between AST and ALT (Pearson correlation coefficient r = .89, *p*<0.001) and strong associations of both markers and natural death in the unadjusted analyses.

Additionally, previously published literature [9] revealed that AST and ALT levels were highly associated with hepatitis B, and with alcohol use disorders as well. Therefore, hepatitis B surface antigen and alcohol use disorders were required as the covariates in the final multivariable models. For easier-to-interpret findings and clinical application, we categorized AST and ALT levels into three subgroups (<40 U/L: normal, 40–80 U/L: mildly elevated, >80 U/L: markedly elevated) and conducted further analyses. The normal upper limit of each marker was set as 40 U/L.

As shown in **Table 3**, mildly elevated AST increased the risk of natural death (odds ratio = 4.37, *p*<0.01) relative to normal AST levels; the risk was even higher when AST was markedly elevated (odds ratio = 53.35, *p*<0.001). Mildly and markedly elevated ALT levels also raised the risks of natural death (odds ratio = 5.11, *p*<0.05; odds ratio = 7.65, *p*<0.001). Additionally, in both of models, comorbid alcohol use disorder was associated with an increased risk of natural deaths, whereas prescription of antipsychotic drugs lowered the risk.

**Table 3:** Multivariable conditional logistic regression of the factors at the latest admission associated with natural death based on AST and ALT respectively (45 deceased patients, 167 living controls).

	AST		ALT	
	Adjusted odds ratio	95% confidence interval	Adjusted odds ratio	95% confidence interval
Enzyme (U/L)				
Normal (<40)	Reference		Reference	
Mildly elevated (40–80)	4.37**	1.47–12.99	5.11*	1.32–19.80
Markedly elevated (>80)	53.35***	5.05–564.19	7.65***	2.53–23.12
Alcohol use disorders	1.76	0.45–6.82	2.38	0.68–8.33
Presence of hepatitis B surface antigen	0.86	0.32–2.32	1.00	0.40–2.53
Use of antipsychotic drugs (presence/absence)	0.34*	0.13–0.91	0.26**	0.10–0.68

*p<0.05,

**p<0.01,

***p<0.001.

### Cohort study

For validating the associations between AST and ALT and natural deaths, we conducted a further study in the whole cohort (N = 1254) by means of Cox proportional hazards analyses (**Table 4**). After adjusting for covariates, mildly elevated AST increased the risk of natural death (adjusted hazard ratio = 2.66, *p*<0.05) relative to normal AST levels; the risk was even higher when AST was markedly elevated (adjusted hazard ratio = 6.75, *p*<0.001). These results are similar to the findings from the phase I study.

**Table 4:** Cox proportional hazards regression of risk factors at the latest admission for natural death based on AST and ALT (N = 1,254).

Characteristic			Unadjusted		Adjusted^a^	
	N (%)	Natural death, n (%)	Hazard ratio	95% confidence interval	Hazard ratio	95% confidence interval
Model AST (based on AST)						
AST+						
Normal (<40)	838 (78.5)	21 (48.8)	1.00	–	1.00	–
Mild elevated (40–80)	160 (15.0)	11 (25.6)	3.37**	1.62–7.00	2.66*	1.21–5.82
Marked elevated (>80)	69 (6.5)	11 (25.6)	8.72***	4.17–18.25	6.75***	3.02–15.11
Alcohol use disorder (yes/no)	114 (9.1)	13 (27.1)	5.42***	2.84–10.34	3.46**	1.64–7.31
Antipsychotic drug use (yes/no)	743 (59.3)	21 (43.8)	0.58	0.33–1.03	0.70	0.37–1.31
HBsAg (yes/no)+++	180 (18.3)	11 (26.2)	1.48	0.74–2.95	1.23	0.60–2.53
Model ALT						
ALT++						
Normal (<40)	814 (76.8)	23 (52.3)	1.00	–	1.00	–
Mild elevated (40–80)	153 (14.4)	8 (18.2)	2.06	0.92–4.61	1.44	0.60–3.43
Marked elevated (>80)	93 (8.8)	13 (29.5)	5.90***	2.96–11.75	5.41***	2.60–11.29
Alcohol use disorder (yes/no)	114 (9.1)	13 (27.1)	5.42***	2.84–10.34	4.35***	2.14–8.83
Antipsychotic drug use (yes/no)	743 (59.3)	21 (43.8)	0.58	0.33–1.03	0.65	0.35–1.22
HBsAg (yes/no)+++	180 (18.3)	11 (26.2)	1.48	0.74–2.95	1.61	0.78–3.33

HBsAg: hepatitis B surface antigen;

a Adjusted for gender, age, hepatitis B surface antigen, antipsychotic drug use, and alcohol use disorder.

*p<0.05,

**p<0.01,

***p<0.001.

+ missing values: 187,

++ missing values: 194,

+++ missing values: 273.

As for the ALT model, only markedly elevated ALT levels raised the risks of natural death (adjusted hazard ratio = 5.41, *p*<0.001). In both models, comorbid alcohol use disorder was associated with an increased risk of natural death. Although the prescription of antipsychotic drugs tended to lower this risk, the reduction did not reach statistical significance.

**Figure 1:**
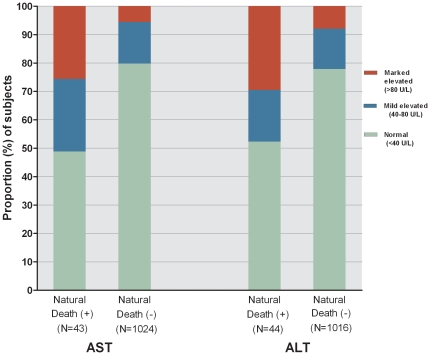
Stacked bar chart of the distributions (%) of aspartate (AST) and alanine (ALT) aminotransferase levels at the latest hospital admission among the cases of natural death and the remaining non-cases of the original cohort, respectively.

The distribution of AST and ALT levels in the cases of natural-death and the remaining non-cases of the original cohort, respectively, are shown in **Figure 1**, in which cases had higher proportions of both markedly and mildly elevated levels than the remaining cohort subjects.

### Subjects without alcoholism

Since comorbid alcoholism was significantly associated with natural death, we then restricted the subjects to the patients without comorbid alcoholism (35 cases with natural death and 1105 subjects without natural death) and conducted further analysis based on the model AST, which revealed similar results. The adjusted hazard ratios for those with mildly and markedly elevated AST levels were 3.19 (95% CI, 1.37–7.41, *p* = 0.007) and 5.20 (95% CI, 1.72–15.74, *p* = 0.004), respectively, without changing direction compared to the model AST listed in **Table 4**.

If based on the ALT model, the hazard ratios for those with mildly and markedly elevated levels were 2.08 (95% CI, 0.82–5.29, *p* = 0.125) and 4.29 (95% CI, 1.72–10.69, *p* = 0.002), respectively.

## Discussion

To our knowledge, this is the first study to identify the factors associated with natural death among patients with methamphetamine dependence. We utilized a national identification system that allowed effective tracking of mortality, except for rare instances of people who migrated outside of the country during the study period (about 2 of 1,000 population per year nationwide [14]). Additionally, through a two-phase study design, we explored the potential factors associated with natural death by means of a nested case-control study and then confirmed the results through a cohort study, which diminished the potential for selection bias. This study provides a clear temporal sequence for investigating the factors associated with natural death, which are critical to the development of intervention strategies.

One of the important findings in this study was the confirmation of the convincing association between AST and ALT elevations and natural death. It provides support for a dose-dependent relationship in which the underlying causes of high AST could induce natural death. As for ALT, the data are similar despite no significantly higher risk in those with mildly abnormal ALT. Thus, both markers, as identified surrogate markers, should be monitored in routine clinical examinations. ALT is an enzyme more specific to liver damage. AST is similar to ALT in that it is another enzyme associated with liver parenchymal cells, but AST is also present in several other organ systems, including cardiac muscle, skeletal muscle, kidneys, and brain tissue [9]. This could mean AST, an enzyme indicating the extent of harm to multiple organs systems (not limited to the liver), is more sensitive than ALT as a predictor of the risk of natural death.

Furthermore, the presence of strong residual effects (comorbid alcoholism-adjusted) of AST/ALT elevations on natural deaths in modeling statistics indicate that methamphetamine could have a direct effect on the organ systems leading to death that is attributed to illness in that organ system. The possible mechanisms of methamphetamine toxicity in humans are difficult to determine from experimental studies due to the illicit nature of methamphetamine and ethical constraints. However, the findings of animal studies could help shed light on the harm of methamphetamine in humans. In rats, a study [15] investigated transaminase activity in response to persistent 4-week administration of amphetamine sulfate and revealed marked increases in serum AST levels and mild increases in ALT levels. In dogs, amphetamine increased plasma levels of both AST and ALT [16], although a later study reported significant increases in AST level, but no change in ALT level [17]. The increase in plasma AST and ALT levels could reflect an effect of amphetamine on the plasma membrane of cells in the organ [16].

The literature depicts several comorbid illnesses [3], [7] methamphetamine users are likely to have that could contribute to death. In our study, comorbid alcohol use disorder was associated significantly with natural death in the AST and ALT models. The relationship between methamphetamine and natural death could be partially mediated by alcoholism. Our results suggest that methamphetamine dependents with comorbid alcoholism comprise a high-risk group for natural death; thus, effective treatment of alcoholism is warranted.

While the factor of comorbid alcohol use disorder was extracted in the phase I nested case-control study, we confirmed the increasing risk of alcohol use disorder for natural death in the phase II cohort study comprising 1254 patients. Additionally, in the phase II study, we conducted survival analyses for the variables at the latest admission instead of at the index admission, which decreased the possibility of selection bias.

The duration of methamphetamine use, intriguingly, was not associated with natural death in this study. In light of a review [8] reporting that the risk of cardiovascular pathology was unlikely to be limited to the duration of methamphetamine use, we suggest that the patients with markedly elevated AST/ALT levels could be individuals who are specifically vulnerable to methamphetamine damage. Additionally, a prior study reported that methamphetamine toxicity is increased with comorbid alcohol use, thus, the two could have a synergistic effect when used together [18]. Nonetheless, we could not precisely evaluate the effect of the interaction between duration of methamphetamine use and alcohol use disorder on the risk of natural death due to the unavailability of information regarding the exact cumulative years of exposure to methamphetamine between the index admission and natural death. Further research is needed to clarify whether methamphetamine and alcohol use disorder have a synergistic effect on the risk of natural death.

The administration of antipsychotic drugs was identified as a potential protective factor for natural death in the phase I study, but it was not confirmed with a significant association in the phase II cohort study. This is an interesting issue for future work.

Methamphetamine users are a high-risk group for hepatitis C virus comorbidity, which is associated with elevations of AST and ALT [7]. In this study, antibody against hepatitis C virus was included as a routine laboratory item in only part of the study period. The partially available data for hepatitis C antibody in the phase I study revealed no statistical significance between case (1/4, 25%) and control subjects (4/24, 16.7%) (*p* = 0.69). Further study is needed to investigate the contribution of hepatitis C virus to natural deaths of methamphetamine-dependent subjects.

The limitations of this study should be considered when interpreting the results. First, this study did not have post-discharge information, in particular the uncertainty regarding methamphetamine exposure and potential confounders or mediators of the relationship between methamphetamine and natural death (e.g., poor nutrition, other exposures). Future improvements and challenges for this work include maintaining contact with the methamphetamine-dependent patients. Second, this study investigated the association between methamphetamine use and natural death – that is, deaths coded as physical illness-related, an inherently heterogeneous category. Nonetheless, if we restricted the outcome as those dying from cardiovascular disease (12 cases), for example, and conducted the further analyses, the findings were similar. In the AST model, the adjusted hazard ratios for the mildly and markedly abnormal AST levels at latest admission in association with natural death were 5.74 (*p* = 0.013) and 8.40 (*p* = 0.008) respectively. Fourth, several laboratory indices including lactate dehydrogenase (LDH) and creatine phosphokinase (CPK) were unmeasured in our clinical routine. Further research is needed to clarify the association between these indices and natural death in the specific population. Lastly, there were some missing data for the identified variables, including AST, ALT, and hepatitis B surface antigen. The further analyses revealed no differences for the proportions of missing values in these variables between the natural death cases and other subjects. Therefore, the systematic missing data did not influence the estimates of this study.

In conclusion, this study provides valuable insight into the factors associated with natural death among patients with methamphetamine dependence. AST and ALT are strong surrogate markers associated with increased risk of natural death. Based on these findings, we emphasize the necessity of intensive follow-up for methamphetamine-dependent patients with elevated AST/ALT levels and comorbid alcohol use disorder for preventing excessive natural deaths.
