# Correction: Elevated Aspartate and Alanine Aminotransferase Levels and Natural Death among Patients with Methamphetamine Dependence

**DOI:** 10.1371/annotation/85803bd9-3be6-4450-b70f-cd4104b87817

**Published:** 2012-01-25

**Authors:** Chian-Jue Kuo, Shang-Ying Tsai, Ya-Tang Liao, Yeates Conwell, Wen-Chung Lee, Ming-Chyi Huang, Shih-Ku Lin, Chiao-Chicy Chen, Wei J. Chen

There is an error in the National Science Council grant number. The correct grant number is, "NSC 99-2314-B-532-002-MY2." 

