# Correction: Elevated Aspartate and Alanine Aminotransferase Levels and Natural Death among Patients with Methamphetamine Dependence

**DOI:** 10.1371/annotation/3bca7672-2fca-4a7e-942d-cc1b4aaa7b60

**Published:** 2012-01-25

**Authors:** Chian-Jue Kuo, Shang-Ying Tsai, Ya-Tang Liao, Yeates Conwell, Wen-Chung Lee, Ming-Chyi Huang, Shih-Ku Lin, Chiao-Chicy Chen, Wei J. Chen

There is an error in the second affiliation. The correct text is, "2 Department of General Psychiatry, Taipei City Psychiatric Center, Taipei City Hospital, Taipei, Taiwan."

